# Simulation of Physicochemical and Pharmacokinetic Properties of Vitamin D_3_ and Its Natural Derivatives

**DOI:** 10.3390/ph13080160

**Published:** 2020-07-23

**Authors:** Subrata Deb, Anthony Allen Reeves, Suki Lafortune

**Affiliations:** Department of Pharmaceutical Sciences, College of Pharmacy, Larkin University, Miami, FL 33169, USA; aallen@ularkin.org (A.A.R.); slafortune@myularkin.org (S.L.)

**Keywords:** vitamin D_3_, cytochrome P450, lipophilicity, solubility, pharmacokinetics, physicochemical, transporter

## Abstract

Vitamin D_3_ is an endogenous fat-soluble secosteroid, either biosynthesized in human skin or absorbed from diet and health supplements. Multiple hydroxylation reactions in several tissues including liver and small intestine produce different forms of vitamin D_3_. Low serum vitamin D levels is a global problem which may origin from differential absorption following supplementation. The objective of the present study was to estimate the physicochemical properties, metabolism, transport and pharmacokinetic behavior of vitamin D_3_ derivatives following oral ingestion. GastroPlus software, which is an in silico mechanistically-constructed simulation tool, was used to simulate the physicochemical and pharmacokinetic behavior for twelve vitamin D_3_ derivatives. The Absorption, Distribution, Metabolism, Excretion and Toxicity (ADMET) Predictor and PKPlus modules were employed to derive the relevant parameters from the structural features of the compounds. The majority of the vitamin D_3_ derivatives are lipophilic (log *P* values >5) with poor water solubility which are reflected in the poor predicted bioavailability. The fraction absorbed values for the vitamin D_3_ derivatives were low except for calcitroic acid, 1,23*S*,25-trihydroxy-24-oxo-vitamin D_3_, and (23*S*,25*R*)-1,25-dihydroxyvitamin D_3_-26,23-lactone each being greater than 90% fraction absorbed. Cytochrome P450 3A4 (CYP3A4) is the primary hepatic enzyme along with P-glycoprotein involved in the disposition of the vitamin D derivatives. Lipophilicity and solubility appear to be strongly associated with the oral absorption of the vitamin D_3_ derivatives. Understanding the ADME properties of vitamin D_3_ derivatives with the knowledge of pharmacological potency could influence the identification of pharmacokinetically most acceptable vitamin D_3_ derivative for routine supplementation.

## 1. Introduction

Vitamin D_3_ or cholecalciferol is a steroid-like endogenous fat-soluble substance either biosynthesized in human skin via sunlight or absorbed from diet and health supplements [1]. Vitamin D can be either vitamin D_2_ (ergocalciferol), primarily found in plants, mushroom and yeast, or vitamin D_3_ (cholecalciferol), which is found in mammalians [2]. Vitamin D_3_, which is a secosteroid (structure with a broken steroid ring), is the predominant form found in humans [1]. Because of its endogenous nature, it has several basal body functions as well as therapeutic role at higher doses [3]. The concentration and biological effect relationship of vitamin D follows a U-shaped curve. The deficiency from vitamin D_3_ leads to multitude of syndromes including bone disorders, dysregulation of cellular growth, immune dysfunction and metabolic diseases [4,5,6,7]. In contrast, elevated vitamin D_3_ levels may lead to hypercalcemia, nephrocalcinosis, vascular calcification in chronic kidney disease and arterial stiffness [6,8]. Vitamin D helps regulate the homeostasis of the human body. Interestingly, vitamin D_3_ is considered a prohormone and is the biologically inactive form of vitamin D_3_. The physiological functions of vitamin D_3_ is achieved by its active form 1,25-dihydroxyvitamin D_3_ or calcitriol. Currently, FDA-approved indications of vitamin D_3_ or its derivatives are psoriasis, management of hypocalcemia, secondary hyperparathyroidism in chronic kidney diseases patients, and the off-label use for vitiligo [9].

**Figure 1:**
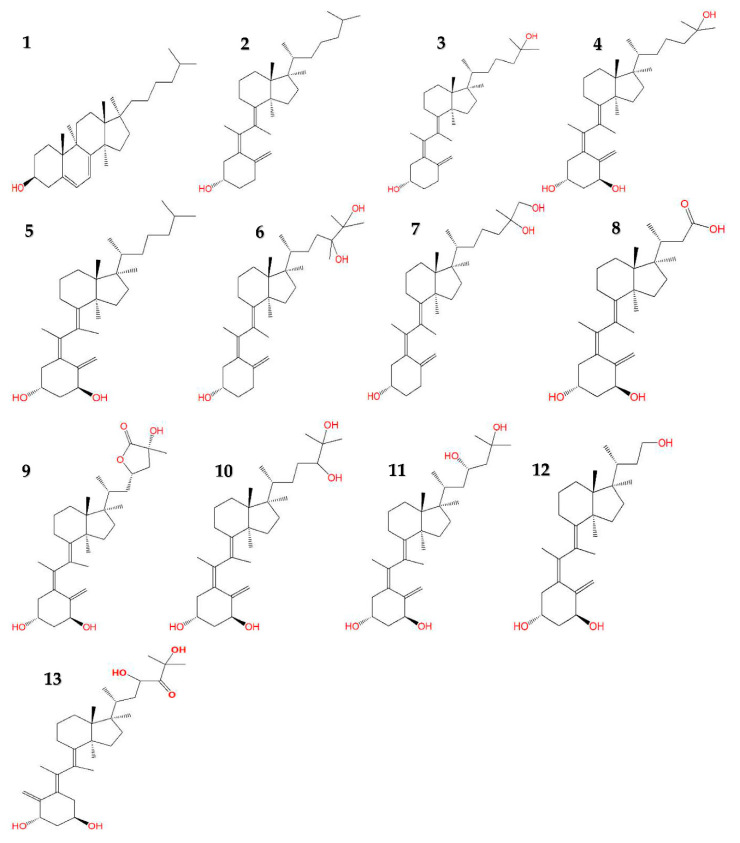
Chemical structures of provitamin D_3_ and vitamin D_3_ derivatives. **1.** Provitamin D_3_ (7-dehydrocholesterol) **2.** Vitamin D_3_ (Cholecalciferol) **3.** 25-hydroxyvitamin D_3_ (Calcifediol) **4.** 1,25-dihydroxyvitamin D_3_ (Calcitriol) **5.** 1-hydroxyvitamin D_3_ (Alfacalcidol) **6.** 24*R*,25-dihydroxyvitamin D_3_ **7.** 25*S*,26-dihydroxyvitamin D_3_ **8.** 1-hydroxy-23-carboxytetranorvitamin D_3_ (Calcitroic acid) **9.** (23*S*,25*R*)-1,25-dihydroxyvitamin D_3_-26,23-lactone **10.** 1,24*R*,25-trihydroxyvitamin D_3_ (Calcitetrol) **11.** 1,23*S*,25-trihydroxyvitamin D_3_ **12.** 1,23-dihydroxy-24,25,26,27-tetranorvitamin D_3_ (Tetranorcholecalciferol) **13.** 1,23*S*,25-trihydroxy-24-oxo-vitamin D_3_. Red letters on structures indicate presence of oxygen-containing functional group(s).

The biosynthesis of vitamin D_3_ and its subsequent conversion to active or inactive metabolites require multiple biochemical reactions. In mammalians, the UV-B radiation from the sunlight converts epidermal 7-dehydrocholesterol (provitamin D_3_) to vitamin D_3_ which is then carried by plasma proteins (e.g., vitamin D-binding protein) to the liver and converted into calcifediol or 25-hydroxyvitamin D_3_ via hydroxylation reaction [3,10]. Though inactive cholecalciferol is the native form, calcifediol is the clinically measured form of vitamin D_3_ in the diagnostic tests and works as a surrogate marker of vitamin D_3_ levels in the human body [10]. According to the Institute of Medicine 2011 report, they recommend a calcifediol serum level of at least 20 ng/mL (50 nmol/liter), though the Endocrine Society Committee emphasizes that at least 30 ng/mL calcifediol serum level is need for basal functions and higher levels required for therapeutic effectiveness [11,12]. The parathyroid hormone promotes the conversion of calcifediol to 1,25-dihydroxyvitamin D_3_ or calcitriol in the kidney through addition of a hydroxy group at carbon-25 [3]. Although calcitriol, the most active form of vitamin D_3_ evaluated so far, is primarily responsible for the health benefits of vitamin D_3_ including bone and anticancer functions, there are several other downstream derivatives in the catabolic pathways of vitamin D_3_ that may function as active vitamin D_3_ metabolite [13]. Hydroxylation and other oxidation reactions of parent vitamin D_3_, calcifediol, and calcitriol produce eight downstream natural metabolites including 24*R*,25-dihydroxyvitamin D_3_, 25*S*,26-dihydroxyvitamin D_3_, calcitroic acid, (23*S*,25*R*)-1,25-dihydroxyvitamin D_3_-26,23-lactone, calcitetrol (1,24*R*,25-trihydroxyvitamin D_3_), 1,23*S*,25-trihydroxyvitamin D_3_, tetranorcholecalciferol (1,23-dihydroxy-24,25,26,27-tetranorvitamin D_3_), and 1,23*S*,25-trihydroxy-24-oxo-vitamin D_3_ (Figure 1) [13,14,15]. Alfacalcidol (1-hydroxyvitamin D_3_) is a synthetic derivatives of vitamin D_3_ [16].

In spite of high levels of supplementation, the serum vitamin D_3_ levels, as indicated through diagnostic laboratory measurements of 25-hydroxyvutamin D_3_, are low, and vitamin D_3_ deficiency has been an epidemic over the last two decades [11,17,18,19]. Typically, soft gel capsules have been the most common form of supplementation available as over the counter agents. However, parenteral and ointment forms of vitamin D_3_ derivatives are also available through prescription [9]. Though there is disagreement between health agencies about what will be a healthy range of vitamin D_3_ to maintain, it is universally accepted that majority of the global population, even in tropical countries, is in the range of vitamin D deficiency [20]. This phenomenon of lack of substantial increase in vitamin D_3_ levels following high amount of supplementation raises serious questions about the absorption, distribution, metabolism and excretion (ADME) of vitamin D_3_ and its derivatives following intake as oral dosage forms.

Physicochemical properties, such partition coefficient, solubility, diffusion coefficient, acid dissociation constant, controls the movement of small molecules through the biological membranes [21]. The partition coefficient is a descriptor of lipophilicity of the molecule and its ability to cross the gut membrane. Though a certain level of lipophilicity is required, a good balance of fat and water solubility is a must to cross the polar-non-polar bilayer membrane in the gut [21]. Similarly, solubility is extremely critical in order to get absorbed in the systemic circulation following oral ingestion and to maintain optimum disposition in central and other compartments. In terms of the ionization status of the molecules, p*K*a is primary for the application of pH partition phenomenon where the p*K*a of the molecule determines what fraction of the drug will be ionized. The ionized species of the drug molecules are unable to cross the gut membrane and typically excreted from the gastrointestinal lumen [21]. The diffusion coefficient, also known as diffusivity, of a molecule is a vital physicochemical property that indicates its ability to cross the biological membrane through passive diffusion [22]. The importance of physicochemical properties remains in the fact that the estimations of pharmacokinetic (PK) parameters, which determine plasma drug concentration and its timeline, is a functionality of physicochemical properties. The parameters such as fraction of dose absorbed and bioavailable, maximum plasma concentration and the time taken to reach to peak concentration, terminal half-life and clearance are indicators of disposition of the small molecule [21,22]. Physicochemical properties and structural features dominate the metabolism and transport profile of the molecules which eventually influence the pharmacokinetic parameters [21,22].

The mass vitamin D_3_ deficiency syndrome and lack of correlation of supplementation and increase in plasma levels necessitates understanding of physicochemical and pharmacokinetic properties of vitamin D_3_ derivatives. Except some limited data on the parent vitamin D_3_ molecule and calcitriol [23,24], the information of physicochemical properties of its downstream metabolites is scant. GastroPlus simulation software is a physiologically-based pharmacokinetics-based simulation software that predicts physicochemical, metabolism, transport and pharmacokinetic behavior of small molecules [25]. The software has been standardized using thousands of prototype molecule and through the use of healthy human physiological conditions including of fasted and non-fasted individuals [25]. Since physicochemical properties play the most critical role in controlling the movement of small molecule chemicals through biological membranes and fluids, understanding the structure-based physicochemical properties and pharmacokinetic profile of vitamin D_3_ derivatives will be important. Thus, the objective of this study was to estimate the physicochemical properties, metabolism, transport and pharmacokinetic parameters and analyze the ADME profile of vitamin D_3_ derivatives using the GastroPlus in silico program. The simulation study of the physicochemical and disposition properties of the vitamin D_3_ derivatives will facilitate the identification and development of the pharmacokinetically optimum compound for supplementation and treatment.

## 2. Results

### 2.1. Physicochemical Properties

The structure-based physicochemical properties of vitamin D_3_ derivatives are listed in Table 1. The molecular weight of vitamin D_3_ derivatives ranged from 360.54 to 446.63 g/mol. The lowest molecular weight vitamin D_3_ derivative was tetranorcholecalciferol, 360.54 g/mol, and the highest was 1,23*S*,25-trihydroxy-24-oxo-vitamin D_3_, 446.63 g/mol. The predicted lipophilicity values obtained through the ADMET Predictor for the twelve vitamin D_3_ derivatives and provitamin D_3_ ranged from 3.0 to 9.02 including calcitriol with a log *P* of 5.5, calcifediol with 6.67, and cholecalciferol has a log *P* value of 8.8. 7-Dehydrocholesterol has the highest lipophilicity of 9.02 and 1,23*S*,25-trihydroxy-24-oxo-vitamin D_3_ had the lowest value of 3. The solubility of vitamin D_3_ compounds ranged from 0.02 to 110 µg/mL. The parent vitamin D_3_ (cholecalciferol) is predicted to have the lowest solubility of 0.02 µg/mL, whereas calcitroic acid has the highest solubility of 110 µg/mL among the analyzed compounds. Similarly, the solubility of calcifediol (25-hydroxyvitamin D_3_) is predicted to be 0.11 µg/mL. Interestingly, the derived diffusion coefficient values are tightly clustered between 0.56 to 0.62 cm^2^/s × 10^−5^ with calcifediol, and calcitriol having the same value of diffusivity. The predicted effective permeability of vitamin D_3_ derivatives had a wide range of 1.82 to 8.14 (cm^2^/s × 10^−4^) with more lipophilic compounds such as provitamin D_3_, cholecalciferol and calcifediol having higher human jejunal effective permeability (*P*_eff_) values of 8.14, 7.93 and 6.41, respectively. In deriving the p*K*a values of vitamin D_3_ compounds, except calcitroic acid, GastroPlus was unable to calculate the descriptor. The MedChem Designer module was employed to calculate the p*K*a which is represented as p*K*a microstate analysis. Microstate p*K*a refers to different protonation states of a chemical structure. Due to the multiple ionizable groups in the vitamin D_3_ structures, different thermodynamic energy state or microstate contributes to the p*K*a of the molecule. As structures undergo metabolism, their p*K*a changes due to the addition of hydroxy groups to the structure to make it more water soluble for excretion [26]. In simulated prediction models analyzing the microstate p*K*a allow for a more accurate assessment of the ionization of multiprotic structures [26]. Except for cholecalciferol, calcitroic acid, and provitamin D_3_, the values listed in Table 1 are the average of two to four microstates. The microstate p*K*a values ranged between 12.88 to 13.34, with the exception of calcitroic acid, which has a predicted p*K*a of 4.96.

### 2.2. Metabolism and Transport Characteristics

The predicted cytochrome P450 (CYP)-mediated metabolism of vitamin D_3_ structures was relatively same as highlighted in Table 2. CYP3A4 is the most associated enzyme with a CYP fraction metabolized (fm) value of 100%. However, calcitroic acid, cholecalciferol and 7-dehydrocholesterol exhibited differential predicted metabolism profile. Calcitroic acid has a predicted CYP fraction metabolism fm 100% with CYP2C9, whereas cholecalciferol is anticipated to be metabolized by two CYP enzymes consisted of CYP2C19 (24.76%) and CYP3A4 (75.24%). 7-dehydrocholesterol metabolism was predicted to be metabolized by CYP2C9 (16.09%), CYP2C19 (17.71%), and CYP3A4 (66.21%). The ADMET simulation did not identify any of the CYP1 to CYP3 family enzymes to be involved in the metabolism of 25*S*,26-dihydroxyvitamin D_3_ and tetranorcholecalciferol.

In the analyses of structure-based prediction of transporters, P-glycoprotein (P-gp) was found to be the primary protein involved with vitamin D_3_ derivatives. Except, calcitroic acid, cholecalciferol and 7-dehydrocholesterol, all the other vitamin D_3_ compounds were substrates of P-gp with an association of 59–91%. Most of the vitamin D_3_ derivatives had high blood-brain barrier penetration except for calcitetrol, 1,23*S*,25-trihydroxyvitamin D_3_, and 1,23*S*,25-trihydroxy-24-oxo-vitamin D_3_.

### 2.3. PK Parameter

The vitamin D_3_ compounds were simulated for pharmacokinetic properties at an oral dose of 100 mg (Table 3). The fraction absorbed percent, which is defined as the percentage of dose that can reach to the intestinal cells, ranged from 0.24–99.95%. The fraction absorbed values for the vitamin D_3_ derivatives were low except for calcitroic acid, 1,23*S*,25-trihydroxy-24-oxo-vitamin D_3_, and (23*S*,25*R*)-1,25-dihydroxyvitamin D_3_-26,23-lactone where each have a predicted fraction absorbed greater than 90%. Cholecalciferol, calcifediol and calcitriol are predicted to have absorption of 0.24%, 2.24% and 8.62%, respectively. Calcitroic acid had the highest fraction absorbed of 99.95%. The observed bioavailability as a percent of dose (*F*%) ranged from 0.2–94.76%. The *F*% of cholecalciferol, calcifediol and calcitriol were 0.2%, 1.78% and 5.44%, respectively. The maximum plasma concentrations (*C*_max_) had a very wide range including 0.58 ng/mL to 3040 ng/mL representing cholecalciferol and calcitroic acid. Similarly, the maximum concentration of vitamin D_3_ compounds that can reach to the liver varied between 0.67 ng/mL and 3480.9 ng/mL. In terms of time taken to reach to the maximum plasma concentration (*T*_max_) spanned between 3.6 to 24 h. Cholecalciferol, the parent vitamin D_3_, is predicted to have a *T*_max_ of 15.28 h, whereas calcitriol and calcifediol is anticipated to have a *T*_max_ values of 5.2 and 4.8 h, respectively. In terms of predicted area under the curve (AUC) extrapolated to infinity AUC_0–∞_ or between 0 to 24 h AUC_0–24_ had the range of 36,318.00–56.42 ng-h/mL or 32,571.00–11.83 ng-h/mL, respectively. In both cases, the highest and lowest values represent AUC_0–∞_ and AUC_0–24_ for cholecalciferol and calcitroic acid. The terminal half-life (*T*_1/2_) values ranged from 1.21 to 7.98 h with 1,23*S*,25-trihydroxyvitamin D_3_ having the lowest *T*_1/2_ of 1.21 h and cholecalciferol has the longest *T*_1/2_ of 7.98 h. Calcitroic acid and 1,23,25-trihydroxyvitamin D_3_ have predicted total body clearance of 2.61 L/h and 49.54 L/h, respectively. Interestingly, simulation of 25*S*,26-dihydroxyvitamin D_3_ and tetranorcholecalciferol did not produce any terminal half-life or total clearance values.

### 2.4. Correlation of Physicochemical and Pharmacokinetic Parameters

The association of major physicochemical properties (e.g., log *P*, solubility, *P*_eff_) and pharmacokinetic parameters (e.g., Fa%, *F*%, *C*_max_, *T*_max_, AUC_0–24,_ *T*_1/2_, CL, C_maxLiver_) were analyzed using correlation studies. The Table 4 depicts the association through coefficient of determination, also known as by the *R*^2^ values, and their interpretation in terms of strength of the correlation. Solubility is correlated with *F*%, *C*_max_ and *C*_maxLiver_ with an *R*^2^ value of >0.75, whereas log *P* was found to be correlated with Fa%, *F*% and *T*_1/2_ with an *R*^2^ value of >0.5. There is a moderate correlation between solubility and Fa% or AUC_0–24_. Between the three physicochemical properties studied for correlation, *P*_eff_ appears to be least associated with the pharmacokinetic parameters. In contrast, solubility is generally strongly correlated with the overall prediction of pharmacokinetic parameters. *T*_max_ and CL appear to be weakly or not correlated with Log *P*, solubility or *P*_eff_.

## 3. Discussion

Vitamin D_3_ is a secosteroid with a wide spectrum of physiological activity and therapeutic functions at concentrations higher than typical endogenous levels [1]. The biological functions of vitamin D_3_ are due to its downstream active metabolites that are produced through hydroxylation and other oxidation reactions. Vitamin D deficiency, as measured through 25-hydrixyvitamin D_3_, is common across the globe and has been the key reason of supplementation in the general population [10,17,18]. However, the correlation of supplementation intake and elevation of plasma levels is not proportionate. Thus, the objective of this study was to predict the physicochemical properties, metabolism, and transport properties that define ADME and PK behaviors of vitamin D_3_ derivatives following oral intake of an immediately release dosage form. Our findings suggest that vitamin D_3_ derivatives have differential physicochemical and pharmacokinetic behavior as they undergo biochemical modifications.

The physicochemical properties were determined by ADMET feature of GastroPlus software. Vitamin D_3_ derivatives included in the present study are predicted to have a wide range of lipophilicity as demonstrated by their octanol/water partition coefficient values. The provitamin D_3_ which is essentially a cholesterol derivative is highly lipophilic, whereas addition of hydroxy group to parent vitamin D_3_ molecule lowered the lipophilicity. The hydroxy groups are known to add to the water solubility or hydrophilic nature of the small molecule. A log *P* value of <5 is considered hydrophilic and >5 is considered hydrophobic. Thus, seven of the thirteen compounds studied appear to be hydrophobic and rest of them hydrophilic. As a comparison, the log *P* of heptane is 4.4 and the predicted log *P* of cholecalciferol, calcifediol and calcitriol is >5.5. The lipophilicity (log *P*) experimental values obtained from the Drugbank database [23] were very similar (average difference 7%) to the values derived from the GastroPlus software. Similarly, the vitamin D_3_ compounds are predicted to have poor water solubility at neutral pH. Compounds with more hydroxy groups offered better water solubility. Except calcitroic acid (p*K*a 4.96), other vitamin D_3_ derivatives studied are strong bases as determined from their p*K*a (approximately 13). Thus, the basic vitamin D_3_ compounds are likely to remain ionized at all pH values of GIT and the moderately weak acid calcitroic will be ionized in intestine. Interestingly, in spite of the molecule known for decades, the experimental data on physicochemical properties of vitamin D_3_ derivatives are limited. The experimental solubility and p*K*a profiles of the studied vitamin D_3_ derivatives are similar to the predicted data [23,24,27,28,29,30]. Literature descriptions of vitamin D_3_ derivatives indicate that these compounds are essentially insoluble in water which necessitates the development of specialized formulations. Similar to the predicted value of this study, calcitroic acid was soluble and (23*S*,25*R*)-1,25-dihydroxyvitamin D_3_-26,23-lactone was sparingly soluble in water, which represented superior solubility profile among the studied compounds [23,24,27,30]. The experimental p*K*a values followed the same trend as the values simulated in the present study [23,29]. The average difference between the predicted and experimental p*K*a values of the compounds is about 2%. It was observed that the predicted solubility of vitamin D_3_ is inversely related to their log *P* values. For example, the parent vitamin D_3_ had the second highest log *P* value (8.8) which corresponded to the lowest solubility (0.02 µg/mL). The other extreme of log *P* value and solubility is appropriate for calcitroic acid. The parent vitamin D_3_ and its mono- or dihydroxy derivatives have the poorest water solubility among the compounds analyzed. However, diffusivity profiles of the vitamin D_3_ derivatives are very similar among the compounds studied in the present work. In contrast, the effective permeability is higher in the compounds with higher log *P* value such as vitamin D_3_, calcifediol and calcitriol. Vitamin D_3_ derivatives with higher number of hydroxy groups, such as calcitroic acid, calcitetrol, are likely to have lower ability to cross the membrane. Overall, vitamin D_3_ derivatives appear to have somewhat unfavorable physicochemical properties when compared with a well-absorbed typical small molecule drug.

Vitamin D_3_ and its derivatives undergoes CYP-mediated metabolism in different human tissues including liver, intestine and kidney [13,31]. The GastroPlus ADMET feature primarily estimate the hepatic metabolism of small molecules. CYP3A4 which is along with CYP3A5 protein represent > 28% of total CYP protein content in the liver and is known to metabolize a vast number of endobiotic and xenobiotic [21]. CYP3A4, CYP2C9 and CYP2C19, in that order, are the enzymes predicted to be involved in the biotransformation of vitamin D_3_ derivatives. CYP3A4 is the main enzyme involved in the hepatic metabolism of all the compound studies, except calcitroic acid, 25*S*,26-dihydroxyvitamin D_3_, and tetranorcholecalciferol. Indeed, the limited CYP-mediated metabolism data available indicate that calcitriol is a substrate of hepatic CYP3A4 [31]. CYP3A4 was also found to be involved in the metabolism of synthetic vitamin D analog such as 1-hydroxyvitamin D_3_ [32]. However, hepatic CYP3A4-mediated biotransformation of majority of the vitamin D_3_ derivatives is still unreported and also the metabolic roles of CYP2C9 and CYP2C19 in vitamin D_3_ disposition need to be explored. Likewise, most of the compounds were the substrates of P-gp efflux protein which suggest that there is a potential for low gastrointestinal absorption following oral ingestion. Currently, there is no experimental report of transport profile and BBB-penetration ability of vitamin D_3_ derivatives. Interestingly, compounds with higher lipophilicity predicted to have high BBB penetration and could offer potential treatment option in the Alzheimer’s or other neurological disorders [4,5]. One of the key limitations in the metabolism predictions remains from the fact that GastroPlus is standardized with hepatic and renal CYP1A2, CYP2A6, CYP2B6, CYP2C8, CYP2C9, CYP2C19, CYP2D6, CYP2E1, and CYP3A4. However, some of the vitamin D_3_ hydroxylation reactions that occur in kidney and other tissues may involve isoforms from endogenous metabolizing family such CYP11, CYP24 and CYP27 [3].

The pharmacokinetic profile of vitamin D_3_ and its derivatives are critical due to the low vitamin D levels in spite of high supplementation pattern across the globe. In the present study, vitamin D_3_ derivatives followed a single compartment pharmacokinetic model. All the studied compounds, except parent vitamin D_3_, 24*R*,25-dihydroxyvitamin D_3_, 25*S*,26-dihydroxyvitamin D_3_ and tetranorcholecalciferol, followed a distinct absorption and elimination phase pattern in 24 h timeline. The other four compounds either showed a broad absorption phase or a linear increase in absorption which eventually plateaued. The fraction of the dose that crosses intestinal cells (Fa%) or becomes systemically available (*F*%) has a wide range with compounds with high lipophilicity are estimated to have very low bioavailability. Compounds with relatively higher water solubility demonstrated better absorption. For example, calcitroic acid with a log *P* of 3.22 and solubility of 110 µg/mL actually predicted to have highest absorption and bioavailability of 99.95% and 94.76%, respectively. In contrast, parent vitamin D_3_ molecule, which has very high lipophilicity and the lowest solubility, has approximately 0.2% absorption and bioavailability. This trend was consistent for the *C*_max_, *T*_max_, and AUC values as well. The elimination descriptors such as terminal half-life and clearance did not follow a certain pattern. For comparative purpose, the pharmacokinetic parameters of vitamin D_3_ derivatives from literature were analyzed. However, the pharmacokinetic experimental data were scarce for the vitamin D_3_ derivatives, except for calcitriol. Similar to the predicted profile, the *C*_max_, *T*_max_, AUC_0–24_, and *T*_1/2_ literature values of oral calcitriol were 10.12 ng/mL, 3.38 h, 95.90 ng-h/mL, and 5.94 h, respectively, and were comparable with the simulated profile [33,34,35]. The pharmacokinetics of the parent vitamin D_3_ were practically not conducive as the human studies focused on the increase in 25(OH)D_3_ concentration following oral administration of the vitamin D_3_ molecules rather than the measurement of parent vitamin D_3_ moiety [36,37]. Other downstream vitamin D_3_ derivatives are yet to be explored for their experimental pharmacokinetic behavior. Since the ability of molecules to diffuse through the gut membrane is a passive process, the other physicochemical properties may not have any significant effect in driving the molecule forward. The molecules with higher number of hydroxy groups, such as 1,23*S*,25-trihydroxy-24-oxo-vitamin D_3_, tetranorcholecalciferol, calcitetrol, (23*S*,25*R*)-1,25-dihydroxyvitamin D_3_-26,23-lactone, and calcitroic acid, can be given in oral dosage form and its gastrointestinal absorption may not be a concern. Gastrointestinal absorption favors hydrophilic over lipophilic substances due to advantageous ionization profile. Except calcitroic acid (moderately weak acid), the vitamin D_3_ compounds analyzed predicted to have high p*K*a (strong base) which suggests that they are likely to remain ionized and poorly absorbed throughout the different parts of GIT. In contrast, calcitroic acid is likely to be unionized in the gastric pH and ionized in the intestine, leading to absorption from the stomach. The normal human stomach has an acidic pH which can range from approximately 1–3. The polar vitamin D_3_ derivatives will be better absorbed in an acidic environment because they have a polar charge. Our correlation studies suggest that lipophilicity and solubility are strongly associated with the pharmacokinetic profiles such as bioavailability and *C*_max_. Though a certain level of lipophilicity is needed to cross the biological membrane, lack of dissolution of drug in the GI fluid is perhaps a major reason of low bioavailability of compounds such as cholecalciferol, calcifediol and calcitriol. Overall, solubility of the vitamin D_3_ derivatives appear to be the rate-limiting step in their absorption.

The changes in vitamin D physicochemical and pharmacokinetic properties are most likely due to the sequential hydroxylation of vitamin D_3_. Hydroxylation is a phase 1 metabolism mechanism that usually produces a chemically stable molecule [38]. It is primarily performed by the human body to make lipophilic substances more water soluble and excretable. Vitamin D has a highly lipophilic structure before its metabolized. When vitamin D undergoes hydroxylation, it becomes more hydrophilic and our data represent this concept by the changes in log *P* and solubility. 7-dehydrocholesterol being the most lipophilic vitamin D_3_ derivative as predicted by GastroPlus is reasonable because it has not undergone metabolism as opposed to 1,23*S*,25-trihydroxy-24-oxo-vitamin D_3_ which is a byproduct of multiple metabolic steps. Calcitroic acid is the product of a series of hydroxylation and oxidation reactions which make it more hydrophilic and highly orally bioavailable. Since CYP3A4 is the hepatic enzyme that metabolizes vitamin D_3_ derivatives, the drugs and natural products, which are inhibitors and inducers of CYP3A4, will likely interfere with the vitamin D_3_ disposition. CYP3A4 has broad active site and is highly susceptible to induction by therapeutic, dietary and environmental agents [39]. These interactions may have the ability to decrease vitamin D activation, increase vitamin D elimination, and subsequently lower serum vitamin D concentration. Similarly, CYP3A4 inducers are also known to increase P-gp transporter expression [40] and can lower the gastrointestinal bioavailability of vitamin D derivatives. For example, various chemotherapy regimens have been found to deplete the vitamin D levels in patients and cause deficiency, which is likely from the interaction of the drugs with vitamin D_3_ disposition [41]. Interindividual differences in the expression and induction profile could be a major factor in differential vitamin D plasma levels observed in the population. Based on the medication and dietary profile of the individual, the induction of CYP3A4 and/or transport may differ, leading to divergent vitamin D plasma profile.

Due to the pleiotropic effects of calcitriol, the most widely studied vitamin D_3_ derivative [1], there is an increased interest in using it and other vitamin D_3_ compounds as therapeutic agents. The parent cholecalciferol, calcifediol and calcitriol have been evaluated for a range of conditions including cancer, inflammation and immunological disorders. However, there is limited information about the pharmacodynamic properties and therapeutic effectiveness of most of the downstream vitamin D metabolites. Since hypercalcemia is a major bottle neck in using calcitriol as a therapeutic agent, the downstream metabolites offer a viable option of reasonable balance between pharmacodynamic and pharmacokinetic properties. For example, the clinical importance of understanding the gastrointestinal absorption and other PK properties of vitamin D remains with its use in oncology, preventing toxicity and subtherapeutic serum levels [13]. It is widely accepted that there is a need to develop cholecalciferol metabolite analogues for the treatment and prevention of cancer [1]. Understanding the physicochemical properties, metabolism, transporter, and pharmacokinetic behavior of the vitamin D compounds will facilitate the selection of vitamin D derivatives that are suitable for GI absorption without exerting hypercalcemic adverse effects.

## 4. Materials and Methods

### 4.1. Vitamin D_3_ Derivative Structures

The structural information were collected on eleven natural vitamin D_3_ derivatives, namely, calcitriol (1,25-dihydroxyvitamin D_3_), 24*R*,25-dihydroxyvitamin D_3_, calcifediol (25-hydroxyvitamin D_3_), 25*S*,26-dihydroxyvitamin D_3_, calcitroic acid (1-hydroxy-23-carboxytetranorvitamin D_3_), vitamin D_3_ (cholecalciferol), (23*S*,25*R*)-1,25-dihydroxyvitamin D_3_-26,23-lactone, calcitetrol (1,24*R*,25-trihydroxyvitamin D_3_), 1,23*S*,25-trihydroxyvitamin D_3_, tetranorcholecalciferol (1,23-dihydroxy-24,25,26,27-tetranorvitamin D_3_), and 1,23*S*,25-trihydroxy-24-oxo-vitamin D_3_. The provitamin D_3_ or 7-dehydrocholesterol (precursor of vitamin D_3_ biosynthesis) and alfacalcidol or 1-hydroxyvitamin D_3_ (a synthetic vitamin D_3_ derivative) were also included in the study for comparative purposes. The Spatial Data File (SDF) format structures were obtained from the PubChem database [24] as the compatible format to upload on the GastroPlus software. The common and IUPAC names of the vitamin D_3_ derivatives were used.

### 4.2. GastroPlus^TM^

GastroPlus software 9.5 version (Simulations Plus Inc., Lancaster, CA, USA) is an in silico mechanistically-constructed simulation tool that can predict the physicochemical, biopharmaceutical and pharmacokinetics properties in humans and animals. The base model of GastroPlus uses backbones of multiple modules including ADMET Predictor, Metabolism and Transporter, and PKPlus. The software offers the option of using several routes of administration including oral, intravenous, ocular, inhalation and dermal. The base module GastroPlus has five different Tabs such as Compound, Gut Physiology, Pharmacokinetics, Simulation and Graph. In the Compound Tab, the SDF structures were imported for creating a “new drug database” and for using the structural properties in Absorption, Distribution, Metabolism, Excretion and Toxicity (ADMET) Predictor and PKPlus modules. The SDF format structures of vitamin D_3_ derivatives were uploaded on the GastroPlus software for analyses in the descriptive modules. In the gut physiology Tab, the standardized software parameters for fasted condition—e.g., gastrointestinal (GI) segment-based pH, transit time, volume, length and radius, CYP3A4 expression and turnover—were used for using in the simulation of pharmacokinetics parameters. The Simulation and Graph Tabs provide the quantitative and visual output in terms of animation and graphical observation.

### 4.3. Estimations of Physicochemical, Biopharmaceutical and Metabolism

We used ADMET Predictor^TM^ feature in GastroPlus software to assess the physicochemical, biopharmaceutical and metabolic properties of the uploaded vitamin D structures. The physicochemical and biopharmaceutical module yielded log *P* (lipophilicity), molecular weight, solubility, diffusion coefficient, p*K*a, and effective permeability. The simulated log *P* values of the vitamin D_3_ derivatives were compared to the literature experimental log *P* value provided by Drugbank database [23]. The CYP-mediated metabolism and fraction metabolized (fm) were predicted based on the structural features. The transporter substrate profile and ability of different vitamin D_3_ derivative to penetrate blood–brain barrier were characterized. In order to use the ADMET Predictor^TM^ feature on GastroPlus software, a “New Drug Database” were created and the SDF format structure files were imported into the database. After each structure file is uploaded an “Import Structure Properties” window pops up. In this window, the “Use Predicted” option was selected to have the ADMET Predictor^TM^ analyses and data for the uploaded structure.

### 4.4. Pharmacokinetic (PK) Analyses

The Pharmacokinetic Tab in the GastroPlus software were used to predict the pharmacokinetic properties of vitamin D_3_ derivatives based on the physicochemical parameters and ADME properties. The compartmental PK model was selected for disposition-related analyses and simulated the bioavailability and elimination under normal human physiology. The Pharmacokinetics Tab included PK parameters, Metabolism/Transporter Scale Factors for liver and gut enzymes, and gut transporters. The characteristics of the virtual subjects used by GastroPlus include American population, 30-year old healthy male with 176.14 cm height, 86.27 kg weight, 24.6% body fat. The standardized default conditions of fasted, and gut physiology were employed analyses. The immediate release tablet dosage form at an initial dose of 100 mg in a dose volume of 250 mL was used in the pharmacokinetic estimations. For each structure various pharmacokinetic parameters were assessed, namely, fraction absorbed (Fa%), bioavailability (*F*%), maximum (or peak) plasma concentration (*C*_max_ µg/mL), maximum concentration in liver (C_max Liver_ µg/mL), time required to maximum plasma concentration (*T*_max_ h), area under the curve from 0 to infinity (AUC_0–∞_) and area under the curve from 0 to 24 h (AUC_0-24_), half-life (*T*_1/2_ h) and clearance (L/h). In the Simulation Tab, the single simulation mode over a length of 24 h was run and the movement of drug after oral administration was followed through an animated gastrointestinal tract including stomach, liver small intestine and large intestine. The single simulation output of the PK parameters was obtained as calculated values.

### 4.5. Correlation Analyses by Microsoft Excel

Microsoft Excel was used to carry out the correlation analyses. Values were plotted for physicochemical properties (log *P*, solubility, *P*_eff_) against pharmacokinetic parameters (Fa%, *F*%, *C*_max_, *T*_max_, AUC_0–24_, *T*_1/2_, CL (L/h), C_maxLiver_ (µg/mL). A linear regression line was extrapolated from the scattered plots to assess the correlation between physicochemical properties and the pharmacokinetic parameters. The association of two types of parameters was determined through coefficient of determination, also known as *R*^2^ values.

## 5. Conclusions

In summary, this is the first report of simulation study reporting the physicochemical properties and ADME behaviors of vitamin D_3_ downstream metabolites. The majority of the vitamin D_3_ derivatives are lipophilic (log *P* values > 5) with poor water solubility which are reflected in the poor bioavailability and other absorption related parameters. CYP3A4 is the primary hepatic enzyme along with P-gp involved in the disposition of the vitamin D derivatives. log *P* and solubility appear to be strongly associated with the GI absorption of the vitamin D_3_ derivatives. The hydrophilic vitamin D_3_ derivatives, such as calcitroic acid, calcitetrol, 1,23*S*,25-trihydroxy-24-oxo-vitamin D_3_, tetranorcholecalciferol, and (23*S*,25*R*)-1,25-dihydroxyvitamin D_3_-26,23-lactone, have significantly better absorption potential than their lipophilic counterparts. Understanding the physicochemical and ADME properties of vitamin D_3_ derivatives with the knowledge of pharmacodynamic profile could influence the identification of pharmacokinetically most acceptable vitamin D_3_ derivative for routine supplementation.

## Figures and Tables

**Table 1:** Estimation of physicochemical properties of provitamin D_3_ (precursor of vitamin D_3_) and vitamin D_3_ derivatives using GastroPlus software. Abbreviations: MW, molecular weight; Diff. Coeff, diffusion coefficient; P_eff_, human jejunal effective permeability.

Compound	log *P*	MW (g/mol)	Solubility (µg/mL)	Diff. Coeff (cm^2^/s × 10^−5^)	P_eff_ (cm/s x 10^-4^)	p*K*a Microstates
Calcitriol (1,25-dihydroxyvitamin D_3_)	5.50	416.65	0.65	0.56	3.45	13.09
24*R*,25-dihydroxyvitamin D_3_	5.17	416.65	0.65	0.56	4.08	13.04
Calcifediol (25-hydroxyvitamin D_3_)	6.67	400.65	0.11	0.56	6.41	12.98
25*S*,26-Dihydroxyvitamin D_3_	5.20	416.65	0.62	0.56	4.25	13.09
Calcitroic acid (1-hydroxy-23-carboxytetranorvitamin D_3_)	3.22	374.52	110.00	0.62	3.61	4.96
Vitamin D_3_ (Cholecalciferol)	8.80	384.65	0.02	0.57	7.93	13.26
Provitamin D_3_ (7-dehydrocholesterol)	9.02	384.65	0.06	0.58	8.14	13.34
Alfacalcidol (1-hydroxyvitamin D_3_)	7.20	400.65	0.08	0.56	4.21	13.32
(23*S*,25*R*)-1,25-dihydroxyvitamin D_3_-26,23-lactone	3.36	444.62	24.30	0.57	2.56	12.91
Calcitetrol (1,24*R*,25-trihydroxyvitamin D_3_)	4.00	432.65	4.61	0.56	2.47	13.10
1,23*S*,25-trihydroxyvitamin D_3_	3.98	432.65	4.77	0.56	2.37	13.27
Tetranorcholecalciferol (1,23-dihydroxy-24,25,26,27-tetranorvitamin D_3_)	3.71	360.54	6.90	0.62	3.65	13.27
1,23*S*,25-trihydroxy-24-oxo-vitamin D_3_	3.00	446.63	62.80	0.56	1.82	12.88

**Table 2:** Cytochrome P450 (CYP)-mediated predicted metabolism and ability to cross blood brain barrier (BBB) of provitamin D_3_ (precursor of vitamin D_3_) and vitamin D_3_ derivatives determined by ADMET Predictor feature of the GastroPlus software. fm, fraction metabolized.

Vitamin D_3_ Derivatives	BBB Penetration	Predicated CYP fm
Calcitriol (1,25-dihydroxyvitamin D_3_)	High	3A4 = 100%
24*R*,25-dihydroxyvitamin D_3_	High	3A4 = 100%
Calcifediol (25-hydroxyvitamin D_3_)	High	3A4 = 100%
25*S*,26-Dihydroxyvitamin D_3_	High	N/A
Calcitroic acid (1-hydroxy-23-carboxytetranorvitamin D_3_)	High	2C9 = 100%
Vitamin D_3_ (Cholecalciferol)	High	2C19 = 24.76%; 3A4 = 75.24%
Provitamin D_3_ (7-dehydrocholesterol)	High	2C9 = 16.09%; 2C19 = 17.71%; 3A4 = 66.21%
Alfacalcidol (1-hydroxyvitamin D_3_)	High	3A4 = 100%
(23*S*,25*R*)-1,25-dihydroxyvitamin D_3_-26,23-lactone	High	3A4 = 100%
Calcitetrol (1,24*R*,25-trihydroxyvitamin D_3_)	Low	3A4 = 100%
1,23*S*,25-trihydroxyvitamin D_3_	Low	3A4 = 100%
Tetranorcholecalciferol (1,23-dihydroxy-24,25,26,27-tetranorvitamin D_3_)	High	N/A
1,23*S*,25-trihydroxy-24-oxo-vitamin D_3_	Low	3A4 = 100%

**Table 3:** Predicted pharmacokinetic parameters of provitamin D_3_ (precursor of vitamin D_3_) and vitamin D_3_ derivatives at a dose of 100 mg. The PKPlus platform was used in a single compartment model.

Compound	Fa%	*F*%	*C*_max_ (ng/mL)	*C*_maxLiver_ (ng/mL)	*T*_max_ (h)	AUC_0-__∞_ (ng-h/mL)	AUC_0-24_ (ng-h/mL)	*T*_1/2_ (h)	CL (L/h)
Calcitriol (1,25-dihydroxyvitamin D_3_)	8.62	5.44	9.86	13.97	5.20	402.48	176.25	2.43	27.58
24*R*,25-dihydroxyvitamin D_3_	8.57	6.76	16.86	20.47	9.76	924.70	340.49	4.30	15.90
Calcifediol (25-hydroxyvitamin D_3_)	2.24	1.78	5.83	7.61	4.80	187.72	96.43	4.98	15.20
25*S*,26-Dihydroxyvitamin D_3_	8.31	8.31	83.16	85.98	24.00	1179.60	1179.60	N/A	N/A
Calcitroic acid (1-hydroxy-23-carboxytetranorvitamin D_3_)	99.95	94.76	3040.00	3480.90	1.92	36318.00	32571.00	6.88	2.61
Vitamin D_3_ (Cholecalciferol)	0.24	0.20	0.58	0.67	15.28	56.42	11.83	7.57	11.15
Provitamin D_3_ (7-dehydrocholesterol)	1.64	1.42	0.59	7.40	5.12	196.05	102.34	7.98	10.21
Alfacalcidol (1-hydroxyvitamin D_3_)	2.03	1.61	6.00	7.91	4.64	152.43	89.59	4.85	15.21
(23*S*,25*R*)-1,25-dihydroxyvitamin D_3_-26,23-lactone	90.15	43.39	105.35	165.31	4.32	1147.60	1146.60	1.45	37.81
Calcitetrol (1,24*R*,25-trihydroxyvitamin D_3_)	37.95	22.58	37.26	53.49	5.36	1312.10	673.27	2.00	30.62
1,23*S*,25-trihydroxyvitamin D_3_	38.45	13.26	15.92	27.24	4.16	475.23	253.95	1.21	49.54
Tetranorcholecalciferol (1,23-dihydroxy-24,25,26,27-tetranorvitamin D_3_)	58.99	58.99	667.36	683.08	24.00	9466.20	9466.20	N/A	N/A
1,23*S*,25-trihydroxy-24-oxo-vitamin D_3_	99.30	52.44	248.32	395.74	3.60	1563.00	1562.50	1.72	33.55

**Table 4:** Correlation of predicted physicochemical properties and pharmacokinetic parameters. Scales of interpretation from coefficient of determination (*R*^2^) values: 0.90–1.00 (strong positive correlation), 0.70–0.89 (fairly strong positive correlation), 0.50–0.69 (moderate positive correlation), 0.10–0.49 (weak positive correlation), 0.09–0.00 (no correlation).

Physicochemical Property	Pharmacokinetics Parameter	*R* ^2^	Interpretation
Log *P*	Fa%	0.66	Moderate positive correlation
Log *P*	*F*%	0.53	Moderate positive correlation
Log *P*	*C*_max_	0.16	Weak positive correlation
Log *P*	*T*_max_	0.01	No correlation
Log *P*	AUC_0–24_	0.16	Weak positive correlation
Log *P*	*T*_1/2_	0.52	Moderate positive correlation
Log *P*	CL (L/h)	0.30	Weak positive correlation
Log *P*	C_maxLiver_ (µg/mL)	0.17	Weak positive correlation
Solubility (µg/mL)	Fa%	0.65	Moderate positive correlation
Solubility (µg/mL)	*F*%	0.75	Fairly strong positive correlation
Solubility (µg/mL)	*C*_max_	0.75	Fairly strong positive correlation
Solubility (µg/mL)	*T*_max_	0.13	Weak positive correlation
Solubility (µg/mL)	AUC_0–24_	0.69	Moderate positive correlation
Solubility (µg/mL)	*T*_1/2_	0.01	No correlation
Solubility (µg/mL)	CL (L/h)	0.04	No correlation
Solubility (µg/mL)	C_maxLiver_ (µg/mL)	0.78	Fairly strong positive correlation
*P*_eff_	Fa%	0.41	Weak positive correlation
*P*_eff_	*F*%	0.23	Weak positive correlation
*P*_eff_	*C*_max_	0.02	No correlation
*P*_eff_	*T*_max_	0.03	No correlation
*P*_eff_	AUC_0–24_	0.02	No correlation
*P*_eff_	*T*_1/2_	0.74	Fairly strong positive correlation
*P*_eff_	CL (L/h)	0.46	Weak positive correlation
*P*_eff_	C_maxLiver_ (µg/mL)	0.03	No correlation
